# A path analysis of multiple neurotoxic chemicals and cognitive functioning in older US adults (NHANES 1999–2002)

**DOI:** 10.1186/s12940-017-0227-3

**Published:** 2017-03-07

**Authors:** Jennifer Przybyla, E. Andres Houseman, Ellen Smit, Molly L. Kile

**Affiliations:** School of Biological and Population Health, College of Public Health and Human Sciences, 101 Milam Hall, Corvallis, OR 97330 USA

**Keywords:** Polychlorinated biphenyls, PCBs, Lead, Cadmium, Cognitive decline, Path analysis, Digit symbol coding test

## Abstract

**Background:**

Polychlorinated biphenyls (PCBs) and metals (lead and cadmium) are neurotoxic and affect neurobehavioral performance. Yet little is known about the association between exposure to multiple neurotoxic compounds and cognitive functioning in older adults.

**Methods:**

Using data from two consecutive cycles of the National Health and Nutrition and Examination Survey (1999–2002), path analysis was used to simultaneously evaluate the association between whole blood concentrations of 14 neurotoxic compounds and cognitive functioning measured by the Digit Symbol Coding Test of the Weschler Adult Intelligence Scale, 3^rd^ Edition in participants 60–84 years of age (*N* = 498). Effect modification was assessed for age (above/below the mean) and sex.

**Results:**

The final path model fit 5 compounds (i.e. PCB 74, PCB 118, PCB 146, PCB 153, and lead). After controlling for co-exposures and confounders, PCB 146 (β = −0.16, 95% CI: −0.29, −0.02, *p* = 0.02) and lead (β = −0.10, 95% CI: −0.20, −0.006, *p* = 0.04) were negatively associated with DSC scores in 60–84 year olds. Whereas, PCB 153 was positively associated with DSC scores (β =0.20, 95% CI: 0.05, 0.35; *p* = 0.01).

**Conclusions:**

This cross-sectional analysis which controlled for collinear exposure to several neurotoxic compounds demonstrated an association between non-dioxin like polychlorinated biphenyl exposure, specifically PCB 146, and lower cognitive functioning, in older adults. Lead exposure was also weakly associated with lower cognitive functioning. Additional studies are needed to determine the causality of the observed associations.

## Background

Exposure to certain industrial chemicals such as lead, cadmium, and polychlorinated biphenyls (PCBs) can affect the central nervous system (CNS) and result in alterations in neurobehavioral performance [1–3]. Once absorbed in the body lead, cadmium, and PCBs can influence brain functioning by affecting neural cells, inducing oxidative stress, and lowering dopamine concentrations [4–6]. Age-related decreases in cognitive functioning is typical as life progresses, mild cognitive impairment (MCI) is a greater than average age-related change in cognitive functioning [7]. Because the US population is rapidly aging and age-related cognitive impairment are predicted to have a high societal and economic impact [8–10], identifying preventable environmental exposures is important to ensure a healthy aging population.

Considerable evidence shows that the developing brain is vulnerable to environmental pollutants [11–15]. There is also evidence that the brain is vulnerable to environmental toxics during the later stages of life due to behavioral, metabolic and physiological changes that occur with aging [16]. These studies are often restricted to examining the effect of single environmental exposures on neurocognitive outcomes in older adults. For instance, the Normative Aging Veterans Affair (VA) cohort demonstrated that blood lead levels were a significant negative predictor of performance on speed memory, spatial copying and vocabulary (*n* = 141) from a battery of 8 cognitive tests [17]. Lead was associated with higher odds of having a Mini Mental State Exam (MMSE) score (*n* = 1,031) less than 24, which is an indication of increased risk of dementia in elderly men [18]. Cadmium also had a negative association with cognitive functioning using Symbol Digit Substitution Test (SDST) in US adults 20–59 (*n* = 5,572) [19]. Whereas another study with Chinese subjects > 65 years of age (*n* = 1,016) observed a negative relationship between cadmium exposure and cognitive functioning using a composite score of the Community Screening Instrument for Dementia (CSID), the Consortium to Establish a Registry for Alzheimer’s Disease (CERAD), Word List Learning Test, the CERAD Word List Recall Test, the Indiana University (IU) Story recall, Animal Fluency test and the IU Token test [20]. Total serum polychlorinated biphenyls (PCBs) have also been associated with decreased measures of memory and learning as measured by the California Verbal Learning Test trial and increase depression as measured by the Beck Depression Inventory in participants (*n* = 253) aged 55–74 years of age living in the vicinity of form capacitor plants in Hudson Falls and Fort Edward, NY [21].

Yet very little is known about how exposure to multiple environmental toxics affect cognition in adults despite the fact that humans are often exposed to mixtures from several different chemical classes. Attempts have been made to examine the effect of PCB mixtures on cognitive functioning, either summing PCB concentrations or using toxic equivalency factors (TEFs). TEFs report the toxicity of a single PCB congener in relation to the most toxic dioxin, 2,3,7,8-TCDD [22]. TEFs are determined using relative effect potencies (REP) established using World Health Organization (WHO) criteria of a compound’s binding capacity and ability to elicit toxic responses from the AhR, persistence in the environment, and accumulation in food chain [23]. Summing concentrations of different PCBs congeners assumes an additive nature of the chemicals specific to the endpoint, which in the case of PCBs and cognitive functioning may or may not be true. Additionally using the TEFs to summarize the potency of PCBs mixtures must be done with caution considering PCBs’ TEFs are both species and response dependent [24].

Therefore, we employed path analysis to examine the association between multiple chemical exposures from different chemical classes on cognitive functioning in older adults. Path analysis is a technique well-suited to modeling multiple environmental exposures because of the ability to determine the magnitude and significance of the relationship between several exposures and an outcome simultaneously while adjusting for multiple comparisons [25, 26]. We hypothesized that exposure to multiple environmental chemicals would be negatively associated with cognitive functioning in older U.S. adults as measured by the Digit Symbol Coding (DSC) test from the Wechsler Adult Intelligence Scale, 3^rd^ edition (WAIS-III).

## Methods

### Study design and population

Data from the National Health and Nutrition Examination Survey (NHANES) continuous cycles 1999–2000 and 2001–2002 were merged for this analysis. NHANES is funded and conducted by the National Center for Health Statistics (NCHS), which is part of the Center for Disease Control (CDC). NHANES is the main tool used to gather data to guide federal health programs and initiatives. NHANES collects data by utilizing physical examinations, specimen collection and surveys to collect data on nutrition and health measurements from a US non-institutionalized representative sample population [27].

This study focused on older adults aged ≥60 years. For confidentiality reasons, NHANES top-codes age at 85 years of age. Therefore, to eliminate outliers due to extreme age and to be consistent with previous studies [28] this sample consists of women and men aged 60–84 years of age who had their blood samples analyzed for lead, cadmium and PCBs and completed the WAIS-III DSC module (*n* = 870). Since having a stroke is a major reason for cognitive dysfunction [29], participants were excluded (*n* = 57) from the analysis if they answered yes to the question “Has a doctor or other health professional ever told you that you had a stroke?”

Of the 813 individuals aged 60–84 year in the subsample that had their blood serum analyzed for PCBs and reported not having a stroke, 715 individuals also had information on DSC scores. However, 102 individuals were missing information on sociodemographic variables with 95, 6, and 1 individuals missing data on poverty income ratio (PIR), smoking status, and education, respectively. Among participants with measured PCB, 115 individuals were excluded due to potential contamination or inadequate biospecimen sample size leaving 498 individuals with complete data [30].

### Exposure

PCBs were measured in blood serum using high-resolution gas chromatography/isotope-dilution. Cadmium and lead were measured in blood samples using inductively coupled plasma mass spectrometry (ICP-MS) [31]. The methods for detecting the environmental chemicals did not change from the 1999–2000 to the 2001–2002 cycles. All analytes were detected at a frequency of 75% or above. For chemical values detected below the limit of detection (LOD), NCHS imputes values equal to the limit of detection divide by the square root of 2 (LOD/√2) into the dataset.

### Cognitive functioning

Cognitive functioning was assessed using the DSC Module of the WAIS-III. Participants were given a key with symbols corresponding to letters. It was ensured the participants had an adequate writing area, glasses (if needed) and could complete the test without distraction before they were allowed to begin the test. Practice sheets were given to ensure the concept of the test was understood before proceeding with the test. Participants were then given the test sheet which showed numbers and asked to draw as many of the corresponding symbols as they could in 120 s. The number of symbols correctly drawn were then summed with a maximum score of 133. Cognitive scores were not provided for participants who refused to take the test, could not complete the test due to distraction, had cognitive or physical limitations, or did not complete the test in the given time limit. Examiners were given extensive instruction on how to score the symbol drawing and 10% of the test were scored twice as quality assurance, all scores were consistent [32]. Cognitive testing procedure did not change from the 1999–2000 to the 2001–2002 cycles.

### Covariates

We explored a variety of sociodemographic covariates based on prior literature showing that they were related to cognitive functioning and/or environmental chemical exposure including: race/ethnicity (Mexican American [MA], Other Hispanic [OH], Non-Hispanic White [NHW], Non-Hispanic Black [NHB]), age (continuous), education level (less than 9^th^ grade, 9-11^th^ grade, high school diploma or GED, some college, college graduate or above), PIR (≤0.99 and ≥ 1.00) and sex (male/female) [28, 33–35]. Since cigarette smoke is a source of cadmium and lead [36] and has been associated with decreased cognitive functioning [37], smoking status was explored as a covariate. Participants with blood cotinine levels ≤10 ng/L were categorized as non-smoking and participants with blood cotinine levels of >10 ng/L were categorized as smokers [38].

### Statistical analysis

All chemical exposures were natural log transformed to address skewness. Descriptive statistics were calculated using a 4 year sampling weight to account for using two consecutive cycles of NHANES using Stata for Windows (version 14, StataCorp LP, http://www.stata.com/). Survey variables including stratification, clustering (PSU) and sample weights corresponding to the 4 year weights of the subsample who had their serum analyzed for PCBs were used to account for the complex sampling design of the NHANES [39].

Path analysis was conducted using MPlus (version 7.4, Muthén and Muthén; http://www.statmodel.com/) to determine the standardized path coefficients, although Stata was used to construct the path diagram. Survey variables and sampling weights were included in the path analysis. The path coefficients in a standardized model can be used to compare the relative influence among variables that have different measurements scales and are similar to beta coefficients in regression. In path analysis the term “effect” refers to statistical effect not causal effect. Complete case analysis was used.

**Fig. 1:**
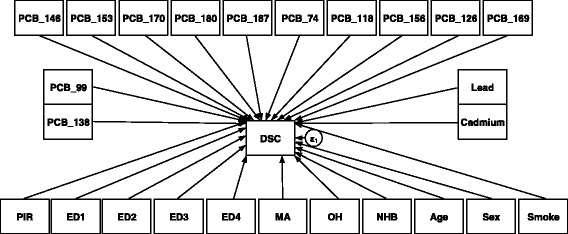
A priori model of 14 neurotoxic compounds and DSC scores and confounding factors. PIR is poverty index ratio, Ed1 is less than 9th grade, Ed2 is High School Grad/GED, Ed3 is Some College or AA degree, Ed4 is College Graduate or above, MA is Mexican American, OH is Other Hispanic and NHB is Non-Hispanic Black

Initially 14 chemicals from the chemical groups of metals (lead and cadmium), non-dioxin like PCBs (PCB 74, PCB 99, PCB 138, PCB 146, PCB 153, PCB 170, PCB 180, PCB 187), and dioxin-like PCBs (PCB 118, PCB 126, PCB 156, PCB 169) were included in the a priori path analysis models (Fig. 1). A more parsimonious model was then fit by removing environmental chemicals that were not significantly (*p*-value ≥ 0.05) associated with cognitive functioning. Models were adjusted for PIR, education, race, age, sex and smoking status. Multiple group analysis along with the Wald Chi-Square test was used to assess effect modification by age (above mean/below mean) and sex because previous studies have reported differences in associations between PCBs and neuropsychological functioning by age and sex [40].

We conducted a sensitivity analysis by re-analyzing the final models using non-lipid adjusted PCBs instead of lipid-adjusted PCBs. This is because the serum samples collected in this study were non-fasting which can influence the concentration of lipophilic compounds [41]. A second sensitivity analysis was conducted to address missingness using Markov Chain Monte Carlo (MCMC) simulations to create 20 complete data sets with imputed values for the missing data.

## Results

The descriptive characteristics of the population are shown in Table 1. We compared the characteristics of the subpopulation included in this analysis with those who were excluded due to missing data. We observed that the individuals included in this analysis were more likely to be Mexican American, Other Hispanic or Non-Hispanic White. Otherwise, the characteristics of the subsample included in this analysis were similar to those with missing data.

**Table 1:** Descriptive characteristics for adults 60–84 years of age who had their blood samples analyzed for PCBs, lead and cadmium and did not have a stroke (NHANES 1999–2002)

Variable	Males and females aged 60–84, who had completed data	Males and females aged 60–84, who did not have complete data	*χ* ^2^ (pvalue)
	N (%^a^)	N (%^a^)	
Age (years)			0.12 (0.73)
60-69	250 (50.24)	162 (54.00)	
70-84	248 (49.76)	153 (46.00)	
Race			8.77 (0.03)
MA^b^	106 (2.98)	76 (.53)	
OH^c^	35 (8.24)	18 (6.78)	
NHW^d^	293 (82.02)	160 (78.97)	
NHB^e^	64 (6.75)	61 (10.71)	
PIR (range)			2.23 (0.14)
1^st^ tertile	166 (28.63)	79 (19.93)	
2^nd^ tertile	167 (32.02)	70 (22.42)	
3^rd^ tertile	165 (39.35)	57 (22.58)	
Missing	NA	109 (35.07)	
Sex			0.12 (0.73)
Male	231 (43.45)	150 (43.00)	
Female	267 (56.55)	165 (57.01)	
Smoking Status			0.08 (0.77)
Smoker (cotinine > 10 ng/L)	413 (81.01)	253 (80.28)	
Non-smoker (cotinine ≤ 10 ng/L)	85 (18.99)	55 (17.87)	
Missing	NA	7 (1.85)	
Education			6.81 (0.15)
Less than 9^th^ Grade	120 (13.09)	91 (12.90)	
9-11^th^ Grade	80 (16.01)	58 (18.70)	
High School Grad/GED	121 (30.25)	63 (25.83)	
Some College or AA degree	96 (21.39)	65 (25.41)	
College Graduate or above	81 (19.26)	37 (16.93)	
Missing	NA	1 (0.22)	
DSC Scores			7.27 (0.06)
1^st^ Quartile	131 (15.88)	56 (10.22)	
2^nd^ Quartile	128 (26.20)	43 (14.88)	
3^rd^ Quartile	129 (27.87)	51 (19.12)	
4^th^ Quartile	110 (30.06)	67 (30.69)	
Missing	NA	98 (25.08)	

^a^Weighted proportion

^b^ *MA* Mexican American

^c^ *OH* Other Hispanic

^d^ *NHW* Non-Hispanic White

^e^ *NHB* Non-Hispanic Black

The mean concentrations for each of the biomarkers are shown in Table 2. The mean concentration of lead and cadmium was 2.17 μg/dL (95% Confidence Interval (95% CI): 2.07, 2.28 μg/dL) and 0.49 μg/dL (95% CI: 0.46, 0.52 μg/dL), respectively. For the lipid adjusted and non-lipid adjusted non-dioxin like PCBs, the highest concentration was for PCB 153 with a geometric mean of 66.99 ng/g (95% CI: 63.66, 70.50 ng/g) and 0.44 ng/g (95% CI: 0.42, 0.47 ng/g), respectively. For the dioxin like PCBs, PCB 118 had the highest lipid adjusted and non-lipid adjusted geometric mean of 20.16 ng/g (95% CI: 18.43, 22.05 ng/g) and 0.13 ng/g (95% CI: 0.12, 0.14 ng/g), respectively.

**Table 2:** Description of the concentration of EDCs including geometric mean, 95% confidence interval (95% CI), percent above LOD, percent who were included in this analysis (NHANES 1999–2002)

Chemical	LOD^a^ (LA^b^/NLA^c^)	Above LOD (%)	Missing (%)	Geometric Mean (95% CI)	Range	Geometric Mean (95% CI)	Range
Metals μg/dL							
Lead (μg/dL)	0.3/NA	100	0	2.17 (2.07, 2.27)	0.4-16.4	NA	NA
Cadmium (μg/L)	0.3/NA	86	0	0.49 (0.46, 0.52)	0.2-4.7	NA	NA
Non-Dioxin Like (ng/g)				Lipid-adjusted		Non-lipid-adjusted	
PCB 74	3.95/0.03	92	0.70	17.78 (16.23, 19.49)	2.8-144	0.12 (0.11, 0.13)	0.02-0.89
PCB 99	2.96/0.03	80	2.10	10.65 (9.88, 11.48)	2.1-132	0.07 (0.07, 0.08)	0.02-0.80
PCB 138	2.96/0.03	92	0.56	44.28 (41.40, 47.37)	2.1-310	0.29 (0.27, 0.31)	0.04-4.01
PCB 146	2.26/0.03	74	1.26	7.86 (7.39, 8.35)	1.6-68.6	0.05 (0.05, 0.05)	0.02-0.69
PCB 153	2.96/0.03	95	0.29	66.99 (63.66, 70.50)	2.1-433	0.44 (0.42, 0.47)	0.02-5.26
PCB 170	2.96/0.03	91	4.76	20.59 (19.76, 21.46)	2.1-129	0.14 (0.13, 0.14)	0.02-1.74
PCB 180	6.20/0.03	97	0.70	51.79 (49.13, 54.60)	4.4-397	0.34 (0.32, 0.36)	0.02-3.65
PCB 187	2.96/0.03	94	0.14	14.93 (14.25, 15.64)	2.1-178	0.10 (0.09, 0.10)	0.02-1.16
Dioxin Like (ng/g)				Lipid-adjusted		Non-lipid-adjusted	
PCB 118	2.96/0.03	93	0.42	20.16 (18.43, 22.05)	2.1-361	0.13 (0.12, 0.14)	0.02-1.95
PCB126^d^	2.54/21.73	75	11.19	33.55 (29.98, 37.55)	1.8-402	223.05 (199.26, 249.68)	15.41-8,188
PCB 156	2.26/0.03	82	1.68	10.14 (9.47, 10.87)	1.6-62.1	0.07 (0.06, 0.07)	0.02-1.03
PCB 169^d^	4.65/32.01	84	11.19	31.66 (29.66, 33.79)	3.3-172	210.45 (197.19, 224.60)	22.7-3,340.4

^a^ *LOD* Limit of detection for lipid adjusted congeners

^b^ *LA* Lipid adjusted

^c^ *NLA* Non-lipid-adjusted

^d^Measured in fg/g

**Fig. 2:**
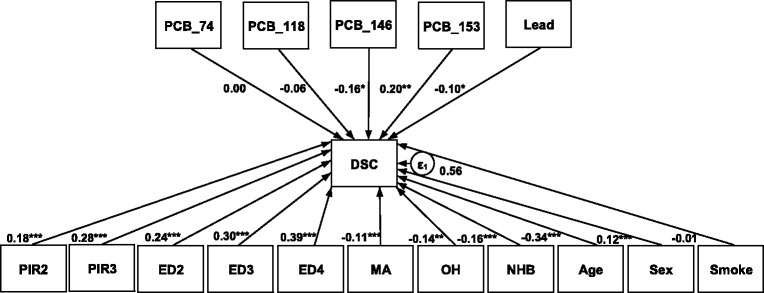
Path analysis of relationship between DSC scores and environmental chemicals after adjusting for confounding factors. Males and females 60–84 years of age, NHANES 1999–2002. Ed1 is less than 9th grade, Ed2 is High School Grad/GED, Ed3 is Some College or AA degree, Ed4 is College Graduate or above, MA is Mexican American, OH is Other Hispanic and NHB is Non-Hispanic Black

Our a priori model included all 14 environmental chemicals in the model. Not all environmental chemicals were significantly associated with cognitive functioning and subsequently dropped from the final path model, which included 5 different chemicals. For all participants (aged 60–84), PCB 146 had the strongest negative association with cognitive functioning scores with a path coefficient of −0.16 (95% CI: −0.29, −0.02) after adjusting for age, sex and smoking status. This effect size can be interpreted as an increase in 1 standard deviation (SD) in the exposure of PCB 146 is associated with a decrease in DSC score of 0.16 points after controlling for other chemical exposures, race, smoking, age and education. Blood lead levels were also associated with a slightly lower DSC score although the strength of this association was weaker (β = −0.10, 95% CI: −0.18, −0.001, *p* = 0.04). We also observed that PCB 153 had a positive association with cognitive functioning scores with a path coefficient of 0.20 (95% CI: 0.05, 0.35) (Fig. 2, Table 3).

**Table 3:** Standardized path coefficients depicting the relationship between 5 chemicals, cognitive functioning and covariates for all participants NHANES 1999–2002 (*N* = 498)

Path	Path coefficient (95% CI)		*p*-value	
	Lipid-adjusted	Non-lipid-adjusted	Lipid-adjusted	Non-lipid-adjusted
PCB 74 → DSC	0.00 (−0.13, 0.13)	−0.001 (−0.14, 0.13)	0.99	0.99
PCB 118 → DSC	−0.06 (−0.20, 0.08)	−0.06 (−0.22, 0.09)	0.41	0.41
PCB 146 → DSC	−0.16 (−0.29, −0.02)	−0.17 (−0.32, −0.02)	0.02	0.03
PCB 153 → DSC	0.20 (0.05, 0.35)	0.21 (0.06, 0.37)	0.01	0.008
Lead → DSC	−0.10 (−0.20, −0.006)	−0.10 (−0.20, −0.007)	0.04	0.04
^a^PIR2 → DSC	0.18 (0.06, 0.30)	0.18 (0.06, 0.30)	0.003	0.003
^b^PIR3 → DSC	0.28 (0.16, 0.41)	0.29 (0.16, 0.41)	<0.001	<0.001
^c^ED2 → DSC	0.24 (0.16, 0.32)	0.26 (0.17, 0.34)	<0.001	<0.001
^d^ED3 → DSC	0.30 (0.22, 0.38)	0.24 (0.16, 0.32)	<0.001	<0.001
^e^ED4 → DSC	0.39 (0.27, 0.52)	0.30 (0.22, 0.38)	<0.001	<0.001
^f^MA → DSC	−0.11 (−0.16, −0.06)	−0.11 (−0.16, −0.06)	<0.001	<0.001
^g^OH → DSC	−0.14 (−0.23, −0.06)	−0.14 (−0.23, −0.06)	0.001	0.001
^h^NHB → DSC	−0.16 (−0.23, −0.10)	−0.16 (−0.22, −0.10)	<0.001	<0.001
Age → DSC	−0.31 (−0.39, −0.24)	−0.31 (−0.39, −0.24)	<0.001	<0.001
Sex → DSC	0.12 (0.03, 0.20)	0.12 (0.03, 0.10)	0.007	0.007
Smoke → DSC	−0.01 (−0.12, 0.09)	−0.01 (−0.12, 0.10)	0.85	0.85

^a^ *PIR2* 2^nd^ Tertile Poverty Index Ratio

^b^ *PIR2* 3 ^nd^ Tertile Poverty Index Ratio

^c^ *Ed2* High School Grad/GED

^d^ *Ed3* Some College or AA degree

^e^ *Ed4* College Graduate or above

^f^ *MA* Mexican American

^g^ *OH* Other Hispanic

^h^ *NHB* Non-Hispanic Black

When analyzing the population stratified above and below the median age, the directions of the associations for PCB 146, lead, and PCB 153 were similar and although the magnitudes of associations between age groups were different (>10%) the strength of the associations were no longer significant (Table 4). The directions of associations were also similar when analyzing the population stratified by sex, although the magnitudes of associations between sexes were different (>10%), specifically every neurotoxic compound had a greater magnitude of association for females in comparison with males, except for PCB 74. However only PCB 153 was significantly associated with cognitive functioning in females (Table 5). No significant interactions were observed for either sex or age (Table 6). Additionally, no substantial differences in the observed associations when PCBs were modeled using non-lipid adjusted PCBs (Tables 3, 4, and 5). The estimates generated when using multiple imputation for missing data were similar to estimates using complete case analysis. Because the models were saturated model fit is perfect with a Tucker Lewis Index (TLI) and Comparative Fit Index (CFI) of 1.00 and Root Mean Square Error of Approximation (RMSEA) and Standardized Root Mean Square Residual (RMSR) of 0.00.

**Table 4:** Standardized path coefficients depicting the relationship between 5 chemicals, cognitive functioning and covariates for participants 60–69 and 70–84 years old, NHANES 1999-2002

Path	Path coefficient (95% CI) *p*-value			
	Lipid-adjusted	Non-lipid-adjusted	Lipid-adjusted	Non-lipid-adjusted
Age 60-69				
PCB 74 → DSC	0.01 (−0.21, 0.23)	0.001 (−0.23, 0.23)	0.92	0.99
PCB 118 → DSC	−0.10 (−0.23, 0.25)	0.02 (−0.23, 0.27)	0.93	0.89
PCB 146 → DSC	−0.20 (−0.43, 0.02)	−0.21 (−0.44, 0.03)	0.08	0.08
PCB 153 → DSC	0.28 (0.03, 0.53)	0.28 (0.02, 0.54)	0.03	0.04
Lead → DSC	−0.13 (−0.28, 0.01)	−0.13 (−0.28, 0.02)	0.07	0.08
^a^PIR2 → DSC	0.16 (0.03, 0.30)	0.16 (0.03, 0.29)	0.02	0.02
^b^PIR3 → DSC	0.33 (0.15, 0.50)	0.33 (0.15, 0.50)	<0.001	<0.001
^c^ED2 → DSC	0.25 (0.08, 0.42)	0.25 (0.08, 0.42)	0.004	0.003
^d^ED3 → DSC	0.28 (0.17, 0.39)	0.28 (0.17, 0.39)	<0.001	<0.001
^e^ED4 → DSC	0.29 (0.18, 0.40)	0.29 (0.18, 0.41)	<0.001	<0.001
^f^MA → DSC	−0.12 (−0.20, −0.04)	−0.12 (−0.20, −0.05)	0.003	0.002
^g^OH → DSC	−0.12 (−0.26, 0.02)	−0.12 (−0.26, −0.02)	0.09	0.09
^h^NHB → DSC	−0.18 (−0.25, −0.10)	−0.18 (−0.25, −0.10)	<0.001	<0.001
Age → DSC	−0.13 (−0.24, −0.01)	−0.13 (−0.24, −0.01)	0.03	0.03
Sex → DSC	0.14 (0.05, 0.24)	0.14 (0.04, 0.23)	0.004	0.005
Smoke → DSC	0.04 (−0.11, 0.19)	0.04 (−0.11, 0.19)	0.58	0.60
Age 70–84 (*N* = 248)				
PCB 74 → DSC	−0.02 (−0.21, 0.18)	−0.02 (−0.22, 0.18)	0.88	0.88
PCB 118 → DSC	−0.13 (−0.29, 0.03)	−0.13 (−0.31, 0.04)	0.12	0.13
PCB 146 → DSC	−0.10 (−0.41, 0.21)	−0.10 (−0.44, 0.23)	0.53	0.55
PCB 153 → DSC	0.12 (−0.14, 0.38)	0.13 (−0.16, 0.42)	0.37	0.40
Lead → DSC	−0.08 (−0.20, 0.04)	−0.08 (−0.20, 0.04)	0.19	0.17
PIR2 → DSC	0.19 (0.03, 0.35)	0.19 (0.02, 0.35)	0.02	0.03
PIR3 → DSC	0.26 (0.11, 0.42)	0.26 (0.10, 0.41)	0.001	0.001
ED2 → DSC	0.25 (0.12, 0.39)	0.25 (0.12, 0.39)	<0.001	<0.001
ED3 → DSC	0.18 (0.04, 0.32)	0.18 (0.03, 0.32)	0.01	0.02
ED4 → DSC	0.30 (0.18, 0.42)	0.31 (0.19, 0.42)	<0.001	<0.001
MA → DSC	−0.11 (−0.18, −0.05)	−0.11 (−0.17, −0.05)	<0.001	<0.001
OH → DSC	−0.20 (−0.32, −0.09)	−0.20 (−0.31, −0.09)	<0.001	<0.001
NHB → DSC	−0.17 (−0.27, −0.07)	−0.18 (−0.27, −0.08)	<0.001	<0.001
Age → DSC	−0.22 (−0.30, −0.10)	−0.20 (−0.30, −0.10)	<0.001	<0.001
Sex → DSC	0.10 (−0.04, 0.24)	0.11 (−0.04, 0.25)	0.15	0.15
Smoke → DSC	−0.06 (−0.18, 0.07)	−0.06 (−0.18, 0.07)	0.35	0.37

^a^ *PIR2* 2^nd^ Tertile Poverty Index Ratio

^b^ *PIR2* 3 ^nd^ Tertile Poverty Index Ratio

^c^ *Ed2* High School Grad/GED

^d^ *Ed3* Some College or AA degree

^e^ *Ed4* College Graduate or above

^f^ *MA* Mexican American

^g^ *OH* Other Hispanic

^h^ *NHB* Non-Hispanic Black

**Table 5:** Standardized path coefficients depicting the relationship between 5 chemicals, cognitive functioning and covariates for females and males age years old, NHANES 1999-2002

Path	Path coefficient (95% CI) *p*-value			
	Lipid-adjusted	Non-lipid-adjusted	Lipid-adjusted	Non-lipid-adjusted
Males (*N* = 231)				
PCB 74 → DSC	0.03 (−0.11, 0.17)	0.03 (−0.13, 0.18)	0.66	0.74
PCB 118 → DSC	−0.01 (−0.25, 0.22)	−0.002 (−0.26, 0.26)	0.92	0.99
PCB 146 → DSC	−0.09 (−0.31, 0.13)	−0.11 (−0.36, 0.14)	0.42	0.38
PCB 153 → DSC	0.05 (−0.22, 0.32)	0.05 (−0.25, 0.35)	0.72	0.74
Lead → DSC	−0.09 (−0.24, 0.06)	−0.09 (−0.23, 0.06)	0.23	0.23
^a^PIR2 → DSC	0.21 (0.05, 0.36)	0.21 (0.06, 0.36)	0.01	0.008
^b^PIR3 → DSC	0.39 (0.21, 0.58)	0.39 (0.21, 0.58)	<0.001	<0.001
^c^ED2 → DSC	0.20 (0.10, 0.30)	0.20 (0.11, 0.30)	<0.001	<0.001
^d^ED3 → DSC	0.25 (0.15, 0.36)	0.25 (0.14, 0.36)	<0.001	<0.001
^e^ED4 → DSC	0.35 (0.20, 0.50)	0.35 (0.20, 0.50)	<0.001	<0.001
^f^MA → DSC	−0.10 (−0.16, −0.03)	−0.10 (−0.16, −0.03)	0.003	0.002
^g^OH → DSC	−0.17 (−0.35, 0.02)	−0.17 (−0.36, 0.02)	0.09	0.08
^h^NHB → DSC	−0.14 (−0.23, −0.05)	−0.14 (−0.23, −0.05)	0.003	0.002
Age → DSC	−0.27 (−0.36, −0.18)	−0.27 (−0.36, −0.18)	<0.001	<0.001
Smoke → DSC	0.05 (−0.10, 0.20)	0.05 (−0.10, 0.21)	0.51	0.51
Females (*N* = 267)				
PCB 74 → DSC	0.005 (−0.25, 0.26)	0.008 (−0.24, 0.26)	0.97	0.95
PCB 118 → DSC	−0.12 (−0.32, 0.08)	−0.12 (−0.33, 0.08)	0.25	0.24
PCB 146 → DSC	−0.21 (−0.45, 0.03)	−0.22 (−0.46, 0.03)	0.08	0.08
PCB 153 → DSC	0.32 (0.05, 0.59)	0.33 (0.05, 0.61)	0.02	0.02
Lead → DSC	−0.12 (−0.26, 0.01)	−0.12 (−0.26, 0.01)	0.08	0.05
PIR2 → DSC	0.15 (0.000, 0.31)	0.16 (0.002, 0.31)	0.05	<0.001
PIR3 → DSC	0.19 (0.03, 0.34)	0.19 (0.03, 0.34)	0.02	0.0001
ED2 → DSC	0.28 (0.15, 0.41)	0.28 (0.15, 0.41)	<0.001	<0.0001
ED3 → DSC	0.23 (0.09, 0.36)	0.23 (0.09, 0.36)	0.001	<0.001
ED4 → DSC	0.22 (0.11, 0.33)	0.22 (0.11, 0.33)	<0.001	0.02
MA → DSC	−0.13 (−0.19, −0.06)	−0.13 (−0.19, −0.06)	<0.001	<0.001
OH → DSC	−0.15 (−0.27, −0.03)	−0.15 (−0.27, −0.03)	0.02	0.11
NHB → DSC	−0.19 (−0.27, −0.11)	−0.19 (−0.27, −0.11)	<0.001	<0.001
Age → DSC	−0.37 (−0.48, −0.25)	−0.37 (−0.49, −0.25)	<0.001	<0.001
Smoke → DSC	−0.08 (−0.22, 0.06)	−0.08 (−0.22, 0.06)	0.28	0.28

^a^ *PIR2* 2^nd^ Tertile Poverty Index Ratio

^b^ *PIR3* 3^nd^ Tertile Poverty Index Ratio

^c^ *Ed2* High School Grad/GED

^d^ *Ed3* Some College or AA degree

^e^ *Ed4* College Graduate or above

^f^ *MA* Mexican American

^g^ *OH* Other Hispanic

^h^ *NHB* Non-Hispanic Black

**Table 6:** Wald’s Chi-Square Test Statistics and *p*-values testing equality of regression coefficients for chemical exposure and cognitive functioning between groups, NHANES 1999-2002

Path	Walds Test Statistic	*p*-value	Walds Test Statistic	*p*-value
	Gender (male/female)		Age (above/below mean)	
PCB 74 → DSC	0.01	0.91	0.08	0.78
PCB 118 → DSC	0.56	0.46	0.99	0.32
PCB 146 → DSC	0.62	0.43	0.44	0.51
PCB 153 → DSC	1.93	0.16	0.51	0.47
Lead → DSC	0.26	0.61	0.37	0.54

## Discussion

In this cross sectional study of the U.S. population we observed that chemical exposures were associated with cognitive functioning in 60–84 year olds. After controlling for co-exposures, we identified PCB 146 as having a negative relationship with cognitive functioning. This is important because non-coplanar PCBs do not activate the arylhydrocarbon receptor (AhR) [11] and subsequently are less of a concern to regulatory bodies. We also observed that lead was weakly associated with lower cognitive scores in this older adult population which is not surprising given that lead is a known neurotoxic compound [42, 43]. This association has also been demonstrated in previous studies [17, 18, 44–46]. Lead can pass through the blood–brain barrier (BBB) by substituting for calcium ions and affect the brain through oxidative stress, altering neurotransmission, or inducing neural cells cell death [3–6]. There is also evidence from mouse studies that exposure to lead results in the accumulation of amyloid beta protein [47], which accumulates in the brain of Alzheimer’s patients [48]. Interestingly, when we were building our model, lead was very strongly associated with lower cognitive scores until we controlled for smoking status which attenuated the strength of the association. This was not unexpected as tobacco smoke is a source of lead and has been shown to be a predictor of cognitive decline [3, 36, 49].

Surprisingly, we did not find an association between cadmium and cognitive functioning despite cadmium being known neurotoxicant [50]. However, the relationship between cadmium and cognitive function in adults is not clear. For instance, several studies using various tests to assess cognitive functioning have found a negative relationship between cadmium exposure and cognitive functioning [19, 20]. In contrast, a study using the MMSE in Malaysian 60–72 year olds (*n* = 54) found a non-significant positive relationship between cadmium exposure and cognitive functioning [51]. These discrepancies could be due to different effects of cadmium at different ages, controlling for different confounders, or study design. Further research is needed to determine the effect of cadmium on cognitive functioning in adults and the elderly.

Similar to previous studies using toxic equivalents, total PCBs or total dioxin-like PCBs, we found a significant negative relationship between non-dioxin like PCB 146 and cognitive functioning after controlling for co-exposure to four other PCB congeners [21, 28, 40, 52, 53]. Unlike dioxin-like PCBs which activates the aryl hydrocarbon receptor (AhR), non-dioxin like PCBs do not activate the AhR [54]. Instead, epidemiology and animal toxicological studies suggest non-dioxin like PCBs act on the CNS by decreasing dopamine production [55–58].

Interestingly, we also observed that PCB 153 had a significant positive association with cognitive functioning. We are unaware of a biological mechanism that can explain the positive association between PCB 153 and cognitive functioning. Further path analysis examining PCB 153 as the sole exposure variable resulted in a non-significant positive relationship between PCB 153 and cognitive functioning, suggesting the significance of individual exposures may differ than when the compound is examined in a mixture. Additionally, PCB 153 has a less potent Neurotoxic Equivalent (NEQ) than PCB 146. NEQs are determined using REPs that are derived using in vitro experiments with neurotoxic outcomes. NEQs were developed to account for the neurotoxicity of PCB congeners, such as ortho-substituted non-coplanar PCBs, which are not included in the TEF scheme [59]. Similarly to modeling sum PCBs, modeling PCB toxic equivalents could overestimate or underestimate risk of altered cognitive functioning by not addressing PCBs’ neurotoxic mode of action.

There are several strength to this study. Specifically, we were able to simultaneous control for the multiple comparisons of several chemical exposures representing different classes of persistent environmental pollutants and other confounders. However, there are several limitations that are worth mentioning. NHANES is a cross-sectional study which prevents us from understanding the temporality between the exposures and the outcomes. Although, the environmental exposure used in this analysis have relatively long half-lives on the order of months to years [60–62] and therefore represent long-term exposure. Additionally, the DSC scores have proven to be a strong predictor of cognitive functioning even when assessed at only one time point [63]. Also we were unable to account for several important covariates. For instance, data was not available on neurotoxic and neuroprotective factors such as methylmercury [64] and omega-3 fatty acids [65], respectively. NHANES only measures a subset of chemicals in each participant which limits the number of compounds that can be included in this analysis. Therefore, we were unable to model additional chemicals which may play a synergistic or antagonistic role in the association between environmental exposures and cognitive functioning. Finally, findings may not be representative of the general population because the analyzed population had a different racial composition compared to the population that was excluded due to missing data.

## Conclusion

In this sample of older US adults, we observed a dose-depended effect between lower cognitive functioning and non-dioxin like PCBs and metals, specifically PCB 146 and lead. Additional animal toxicity and epidemiology are needed to confirm the role of non-dioxin like PCBs and declines in cognitive function. Continued development and incorporation of the recently proposed NEQs will allow the impact of non-dioxin like PCBs on neurotoxicity to be properly addressed. Since neurodegenerative disease have a high economic and social burden and many environmental exposures are preventable the potential impact of exposure to neurotoxic compounds in late life is great and warrants further investigation to guide prevention and intervention efforts.

## Abbreviations

AhR: Arylhydrocarbon receptor
CDC: Center for disease control
CERAD: Consortium to establish a registry for alzheimer’s disease
CNS: Central nervous system
CSID: Community screening instrument for dementia
DSC: Digit symbol coding
ICP-MS: Inductively coupled plasma mass spectrometry
IU: Indiana University
LOD: Limit of detection
MA: Mexican American
MCI: Mild cognitive impairment
MMSE: Mini mental state exam
NCHS: National Center for Health Statistics
NEQ: Neurotoxic equivalent
NHANES: National Health and Nutrition Examination Survey
NHB: Non-hispanic black
NHW: Non-hispanic white
OH: Other hispanic
PCBs: Polychlorinated biphenyls
PIR: Poverty index ratio
REP: Relative effect potencies
SDST: Symbol digit substitution test
TEFs: Toxic equivalency factors
VA: Veterans affair
WASI-III: Weschler adult intelligence scale, 3^rd^ edition
WHO: World Health Organization
